# Family Functioning and Children’s Post-Traumatic Stress Symptoms in a Referred Sample Exposed to Interparental Violence

**DOI:** 10.1007/s10896-015-9769-8

**Published:** 2015-08-02

**Authors:** Machteld D. Telman, Mathilde M. Overbeek, J. Clasien de Schipper, Francien Lamers-Winkelman, Catrin Finkenauer, Carlo Schuengel

**Affiliations:** Section of Clinical Child and Family Studies, VU University Amsterdam, Van der Boechorststraat 1, 1081 BT, Amsterdam, The Netherlands; EMGO Institute for Health and Care Research, VU University Medical Center, Van der Boechorststraat 7, 1081 BT, Amsterdam, The Netherlands; Child and Youth Trauma Center, Zuiderhoutlaan 12, 2012 PJ, Haarlem, The Netherlands

**Keywords:** Domestic violence, Post-traumatic stress, Family functioning, Child abuse, Neglect, Emotional security, Parenting stress, Maltreatment

## Abstract

This study examined the association between interparental violence (IPV), child abuse and neglect, other traumatic experiences, and children’s post-traumatic stress (PTS) symptoms and explored the moderating role of family functioning in the aftermath of IPV. One hundred and twenty IPV-exposed children (53.3 % male, *M* age = 9.85) and parents who were referred to community mental health centers participated in the study. Combined, IPV, child abuse and neglect, and other traumatic experiences were associated with PTS symptoms. For family functioning, higher levels of parenting stress were associated with higher levels of PTS symptoms. No moderating effects were found. To understand the variability in PTS symptoms among children exposed to IPV, other traumatic and stressful experiences need to be taken into account.

Interparental violence (IPV) is common, with as many as 15.5 million children affected every year, including seven million children who experience severe partner violence in the United States (McDonald et al. 2006). In the Netherlands, for every 1000 children, 12 have witnessed IPV (Euser et al. 2013). Converging evidence suggests that exposure to violence between parents or caregivers in the home is associated with an increased risk of emotional and behavioral problems during childhood and adolescence (Kitzmann et al. 2003; McDonald et al. 2006; Wolfe et al. 2003), as well as poor health outcomes later in life (Dube et al. 2003). Children’s exposure to IPV may consist of a range of distressing events, from direct exposure to verbal aggression or physical fights to seeing parents threatening each other with weapons, or indirect exposure, by learning about the consequences of parental violence through the physical and emotional impact on parents (Holden 2003). Because children’s responses to IPV vary, it is important to identify protective factors and risk factors in order to optimize interventions aimed at preventing or reducing problems in this group.

## Interparental Violence and Post-Traumatic Stress

Among the psychological responses to shocking, upsetting events in children, the most commonly studied is post-traumatic stress disorder (PTSD; Trickey et al. 2012). In addition to the presence of (in) direct exposure or witnessing traumatic event(s), four symptom clusters are characteristic of PTSD: intrusion symptoms; avoidance of traumatic stimuli; negative alterations in cognitions and mood; and changes in arousal and reactivity (American Psychiatric Association 2013). The symptoms should be present for at least 1 month and must cause significant functional impairment in order to meet the diagnosis of PTSD. Regardless of the type of traumatic event, a recent meta-analysis reported that 15.9 % of children and adolescents were diagnosed with PTSD after experiencing traumatic events (Alisic et al. 2014).

While earlier accounts of PTSD have focused on exposure to rare and single traumatic events (e.g., natural disasters), the field has now included more frequently occurring experiences that form a threat to the child or a loved one (American Psychiatric Association 2013). In this context, PTSD after exposure to IPV has received increased attention. Studies have confirmed the heightened occurrence of post-traumatic stress symptoms (PTS), such as avoidance, intrusions, and arousal in children exposed to IPV (Crusto et al. 2010; Graham-Bermann and Levendosky 1998; Kilpatrick and Williams 1998; Lamers-Winkelman et al. 2012; Margolin and Vickerman 2007; Rossman et al. 1997). Although less well studied, the impact of IPV appears stronger for children’s PTS symptoms (*d* = 1.54, 6 studies) than for internalizing problems (*d* = 0.48, 60 studies) or for externalizing problems (*d* = 0.47, 60 studies; Evans et al. 2008). Further, the meta-analysis by Alisic et al. (2014) reported that traumatic experiences of an interpersonal nature, such as IPV, results more often in PTSD, with 1 in 4 exposed children affected, compared to rates of 1 in 10 for those who experienced a non-interpersonal trauma.

Despite the documented negative consequences of IPV on children’s mental health, it is also known that not all children who experience IPV will develop PTS symptoms, and in this regard, estimates of individual studies vary. For example, Spilsbury et al. (2007) reported that 12 % of children reported clinical levels of PTS symptoms. In a Dutch sample, Lamers-Winkelman et al. (2012) found that 57 % of IPV-exposed children who were referred for treatment scored in the clinical range of PTS symptoms. This suggests that there are factors that make children more prone or less prone to developing PTS symptoms after exposure to IPV. It is important to study the factors that are associated with IPV and PTS symptoms in exposed children to better understand their responses and to inform treatments for those who are affected.

To date, research is lacking on identifying trauma characteristics and the conditions by which their effects can vary with respect to children’s trauma symptoms after IPV exposure. The study of trauma symptoms after IPV exposure is important because of two reasons. First, compared to single-event related PTSD, IPV is a multifactorial phenomenon that is frequently associated with other potential traumatic events and abusive experiences (Margolin and Vickerman 2007). Therefore, PTS symptoms may derive from multiple sources. A second reason is that one of the key aspects of IPV traumatic experiences is that they occur between the child’s parents and/or caregivers, who normally should be their sources of support and safety. A further consequence of IPV might be that parents’ resources to cope with the demands of parenthood can be undermined (Holt et al. 2008; Levendosky and Graham-Bermann 2001). Given these complexities, we believe it is necessary to combine perspectives from both trauma research and family research in order to better understand children’s PTS symptoms after exposure to IPV.

## Characteristics of Trauma Exposure in Violent Families

One of the concepts in PTSD research is that the preceding event needs to have enough impact to trigger the disorder. When examining IPV exposure, trauma exposure is often better conceptualized as a series of events rather than a single discrete event (Margolin and Vickerman 2007), which complicates the study of effects of event impact. For example, it is difficult to identify a key traumatic event when exposure is likely to consist of multiple, chronic threats that are difficult to avoid when they occur in the family home. However, we can study the effects of the trauma impact as reflected by the *severity* of IPV and its *chronicity*, for example, by the number of different violent incidents and the duration of the violent relationship. Kitzmann et al. (2003) reported in their meta-analysis of 70 correlational studies that greater IPV exposure was associated with poorer child outcomes (*d* = −0.29). Less is known about the effects of chronicity of IPV on children’s trauma symptoms. One study reported that duration of the abusive relationship increased the chance of children’s exposure to physical and sexual IPV (Vatnar and Bjorkly 2011). However, Lamers-Winkelman et al. (2012) did not find a significant association between IPV duration and trauma symptoms. In this study, we will examine whether severity and chronicity of violence in parental relationships is associated with higher levels of PTS symptoms in children.

Another important characteristic of IPV is that, compared to single event trauma, it rarely occurs in isolation. A growing body of evidence points to the cumulative nature of the traumatic events that children exposed to IPV face. Results from the National Survey of Children’s Exposure to Violence (Finkelhor et al. 2013; Turner et al. 2012) show that child victimization is likely repetitive and cumulative in nature, with many co-occurring risk factors involved that are associated with PTS symptoms. Finkelhor et al. (2007) therefore introduced the term “polyvictimization” for children who are exposed to different kinds of interpersonal trauma, such as sexual abuse, physical abuse, bullying, and exposure to family violence. Others have also stressed the importance of polyvictimization as an important factor in the likelihood that children will experience PTSD (Margolin and Vickerman 2007), given that co-occurrence rates between IPV and child abuse are about 40 % in families referred for clinical care (Appel and Holden 1998).

Further, IPV often co-occurs with other stressful events, such as moving houses, parental divorce, hospitalization of a family member, and incarceration of a parent. A recent study by Graham-Bermann et al. (2012) found that, among young children who were exposed to IPV in the past 2 years, 38 % were also exposed to other stressful events. Children exposed to both IPV and stressful events had higher rates of PTSD than children who were exposed to IPV alone (Graham-Bermann et al. 2012). Given that IPV-exposed children are more likely to experience abuse and other stressful events, we will examine the role of these factors in combination with IPV exposure and their association with PTS symptoms.

## Family Functioning Post-IPV

Family factors have been implicated in children’s adjustment to parental conflict (Cummings and Davies 2011) and recovery from traumatic experiences, such as in child sexual abuse (Corcoran and Pillai 2008). Regardless of type of trauma exposure, the child’s family environment has been identified to play a role in the development of children’s PTS symptoms (Trickey et al. 2012), whether negative or positive. We believe it is important to study negative and positive qualities of family functioning in IPV exposed children when the trauma has occurred in the family system and affects family members and their relationships. For example, the occurrence of IPV can tax the resources available for good parenting. One concept studied in this respect is parenting stress (Crusto et al. 2010), which refers to the negative feelings and stressors experienced to the self as parent and to the child arising from the demands of parenthood (Abidin 1995; Deater-Deckard 1998). Parenting stress is associated with children’s adjustment following IPV (Levendosky and Graham-Bermann 1998; Roberts et al. 2013). For example, Levendosky and Graham-Bermann (1998) found that psychological and physical IPV was associated with mothers’ parenting stress in raising their young children. Further, mothers’ parenting stress was, in turn, associated with their children’s internalizing and externalizing problems. Finally, the effects of IPV on adjustment were conditional on the levels of parenting stress. At high levels of psychological IPV, high parenting stress had more impact on children’s internalizing problems. For externalizing problems, the effects of high parenting stress were most pronounced at low levels of psychological IPV (Levendosky and Graham-Bermann 1998). The significance of parenting stress as a risk factor in IPV-related PTS symptoms in children has only recently been studied. Crusto et al. (2010) found that parenting stress was associated with preschool children’s PTS symptoms. This suggests that parenting stress could shape traumatic responses of children when exposed to IPV. It is at present unknown whether parenting stress could act as a moderator of the association between IPV characteristics and children’s PTS symptoms.

In contrast, the family environment can also provide protective factors in the context of child trauma. Research has highlighted the role of parental support in the aftermath of trauma, because children need to be able to rely on their parent(s) to cope with their emotional distress (Berkowitz et al. 2011). Emotional security refers to the process that a child uses to appraise his or her family as a source of safety and security (Cummings and Davies 2011). When emotional security levels are high, children have the confidence that they can rely on their family members as sources of safety, support, and predictability (Forman and Davies 2005). Studies have shown the importance of all family members in contributing to children’s feelings of emotional security (McCloskey et al. 1995; Miller et al. 2014). With respect to IPV, research has mainly focused on the negative effects of parental conflicts and IPV, that is, emotional *in*security (Davies et al. 2012; Davies et al. 2006; El-Sheikh et al. 2008). It is currently unknown whether child emotional security in the family in the aftermath of IPV can function as a protective factor. When children are able to maintain or rebuild confidence in their family once the IPV has stopped, emotional security could potentially buffer against developing mental health problems such as PTS symptoms. Despite the significance of family factors in the aftermath of trauma, research on these factors in children exposed to IPV is limited, and our understanding of the role of parenting stress and children’s feelings of emotional security is incomplete.

## The Current Study

IPV confers risk for developing PTS symptoms in exposed children, but there is great variability in that some children fare poorly while others seem to adjust well (Alisic et al. 2014). Further, IPV exposure is associated with abuse, neglect, and other traumatic experiences. Although much progress has been made in examining the traumatic effects of IPV on children, little is known about the role of parenting stress and emotional security as moderators of the impact of victimization. Examining associated risk factors and moderating factors for PTS symptoms in the context of IPV is important as this can inform treatment plans (Margolin and Vickerman 2007). Therefore, we first examined the associations between IPV, child abuse and neglect, other potentially traumatic events, and PTS symptoms in IPV-exposed children. Second, we examined whether these associations are moderated by parenting stress and children’s feelings of emotional security as indices of family functioning.

Based on previous research, we hypothesized a positive association between IPV, child abuse and neglect, and other potentially traumatic events and children’s PTS. Given that parenting stress is frequently associated with children’s behavioral and emotional problems, we hypothesized a positive association between parenting stress and children’s PTS, such that more parenting stress was associated with higher levels of symptoms. Because less is known about the relationship between emotional security and children’s PTS, this association was explored in this study and no specific hypothesis was made. Similarly, the moderating roles of parenting stress and emotional security in the association between the above listed indicators of potential traumatic experiences were examined in explorative analyses and no hypotheses were proposed.

## Method

### Participants

This sample is part of a larger randomized controlled trial (RCT) that examined the effect of treatment factors in community-based intervention for 6- to 12-year-old children exposed to IPV and their caregiving parent. Parent–child dyads were included in the study when they had experienced psychological IPV, physical IPV, or both, which had stopped at the time of enrolling in the intervention. The details of this RCT are described elsewhere (Overbeek et al. 2012). From the total sample (*N* = 164), we selected the subsample (*n* = 120) of children aged 8–12 years (*M* age = 9.85, *SD* = 1.12; 64 boys and 56 girls) and their caregiving parent who completed questionnaires before the start of the intervention. This selection was based on the age restriction of the validity of the children’s questionnaire that was used. In the vast majority (94 %), children participated with their biological mother. Furthermore, 68 % of the children came from single-parent households. The ethnical background of families varied: 42 % Dutch, 24 % Turkish/Moroccan, 17 % Antilles/Suriname, and 17 % other. A considerable number of families (63 %) received an annual income below €15.000, which is considered the poverty threshold for a single-parent family with two children in the Netherlands.

### Procedure

The study received ethical approval of the local Medical Ethics Committee (METc VUmc 2009/99/NL26649.029.09). After providing informed consent, parents and children were invited to their community mental health center 1 week prior to the start of the intervention. During a 1.5 h visit, the parent and child completed questionnaires in two separate rooms. Researchers or research assistants were present to provide supervision. Assessments followed a structured format described in a study protocol that was developed for this study, and measures were always administered in the same order. When children had difficulty in understanding any of the questions, researchers or research assistants would answer their questions based on suggestions from the study protocol to ensure comparable administration of the measure. When children had difficulty reading the questions, a researcher or research assistant would read the questions for them, and the children would indicate their answer. Parents received a financial compensation (€15,-), and children received a small gift as a reward for their participation.

### Measures

#### Children’s Trauma Symptoms

Children’s trauma-related symptoms were assessed with the Dutch version of the Trauma Symptom Checklist for Young Children (TSCYC, Tierolf et al. 2013). The 90-item TSCYC is a parent-reported measure of children’s trauma-related symptoms of children between age 3 and 12. We used the PTS-total subscale with T-scores. This subscale consists of 27 items that cover symptoms of intrusions, avoidance, and hyperarousal. Parents rated the occurrence of these symptoms during the past month on a 4-point Likert scale, ranging from 1 (*not at all*) to 4 (*very often*). The original TSCYC questionnaire has been used before with children exposed to violence and has adequate reliability and validity (Briere et al. 2001). The internal consistency of the PTS-total subscale in this study was good (*α* = .90).

#### Severity of IPV Exposure

Parents reported on both their own and their (ex)partners’ acts in their relationship conflicts during the past year of the relationship by completing the Conflict Tactics Scales-2 (CTS2, Straus 2001). For all 20 incidents (eight regarding psychological aggression, 12 regarding physical assault), parents were asked to rate how often they and their (ex)partner engaged in this specific act, ranging from 1 (*never happened*) to 8 (*more than 20 times in the past year*). To create an index of severity of IPV, we calculated the total number of different incidents by both the parent and their (ex)partner that had ever occurred in the relationship, which could range from 0 to 40, as there were two ratings for all 20 incidents. Cronbach’s *α* was .89 for this IPV severity measure.

#### Chronicity of IPV Exposure

In addition to the CTS2, questions regarding the duration of the violent relationship and experienced threat were asked. Chronicity of IPV exposure was calculated using the difference score between first experience of threat as the start date and last experience of threat as the end date. Furthermore, time since IPV exposure was calculated using the difference score between the end date of the experienced threat and date of completion of the questionnaire.

#### Child Abuse and Neglect

To assess child abuse and neglect, we used the Conflict Tactics Scale Parent–child(CTSPC, Straus 2001). This instrument enquires about parental physical assault and psychological aggression towards the child and parental neglect. For each topic about physical assault (eight in total) and psychological aggression (five in total), parents were asked to rate on an 8-point scale how often they and their (ex)partner engaged in this specific act, ranging from 1 (*never happened*) to 8 (*more than 20 times in the past year*). Parents were also asked to rate the occurrence of their own acts of neglect towards their child on similar 8-point scales. Each rating was then included in a total score, indicating whether or not the act had ever occurred. Two composite scores were created that included the sum of ever-occurred incidents of child abuse (26 items, Cronbach’s *α* = .81) and the sum of ever-occurred incidents of child neglect (seven items, Cronbach’s *α* = .62).

#### Other Potentially Traumatic Experiences

To assess other potentially traumatic events (PTEs) in the child’s life, items from the Parent Report of Traumatic Impact (Friedrich 1997) questionnaire were used. This parent-report measure enquires about other potentially traumatic experiences that the child has ever experienced. We calculated a total score from a range of 21 reported life events, such as divorce, hospitalization of the parent, incarceration of a parent, suicide attempt of a family member, changing schools, and moving houses.

#### Children’s Feelings of Emotional Security in the Family

The Security in the Family System scale (Forman and Davies 2005) was used to assess how much children perceived their families as a reliable source of protection, stability, and support. The Secure subscale was used in this study, which assesses a secure pattern of emotional security (e.g., “*I feel I can count on my family to give me help and advice when I need it*”). Children indicated the extent to which they agreed with seven statements using a 4-point scale ranging from 1 (*completely disagree*) to 4 (*completely agree*). Higher scores reflect higher levels of experienced emotional security in the family. In our study, Cronbach’s *α* was .75.

#### Parenting Stress

Parenting stress was assessed with the 25-item short form version of the Parenting Stress Index (PSI, Abidin 1995). Questions on the PSI covered child-related stress as well as stress related to the parent. On a 6-point Likert scale, parents rated whether they agreed or not with statements from 1 (*totally disagree*) to 6 (*totally agree*). Higher scores reflect higher parenting stress. In the current study, internal consistency was high (*α* = .93).

### Statistical Analyses

Descriptive analyses explored the nature of IPV, child abuse and neglect, potentially traumatic experiences, and children’s PTS symptoms in the sample. A zero-order correlation matrix described the associations between IPV, child abuse and neglect and other experiences, family functioning, and PTS symptoms. Hierarchical multiple regression analyses were used to examine the effects of IPV, child abuse and neglect, potential traumatic experiences on children’s PTS symptoms, as well as the moderating effect of family functioning on this association (Aiken and West 1991). All analyses were conducted in IBM SPSS Statistics version 21 (IBM 2012).

## Results

### Descriptive Analyses

#### Experience of IPV

In 76 % of the families, the partner in the violent relationship was the biological parent of the child. While 18 % of parents who were in violent relationships stayed together with their partner, 58 % did not live together anymore but remained in contact. On average, the violence had stopped 0.81 years ago (*SD* = 1.34). With respect to *IPV severity*, the mean number of ever-occurred incidents of IPV was 17.87 (*SD* = 7.29). When examining the two domains of IPV separately, the mean number of ever-occurred physical assaults was 9.07 (*SD* = 4.75) and the mean number of ever-occurred psychological aggressive acts was 8.80 (*SD* = 3.32). The incidence of serious violence was high in our sample, in that 60 % of parents reported having been beaten up by their (ex)partner, 58 % of parents reported that their (ex)partner had tried to strangle them, and 35 % of parents reported that their (ex)partner had used a gun or knife against them. Finally, *IPV chronicity* varied considerably in our sample (*M* = 7.83 years, *SD* = 5.93, range: 0.04–29.28 years). More than 50 % of the children were exposed to violent relationships that lasted eight years or longer.

#### Child Abuse and Neglect

The mean number of ever-occurred incidences of child abuse was 4.33 (*SD* = 3.74). More than 50 % of the parents reported three or more incidents of child abuse. For neglect, the mean number was 1 (*SD* = 1.30); the total reported experiences of neglect ranged from one to six. The most frequently rated (25 %) form of neglect was parents’ reduced ability to show or tell their children they loved them because of being caught up with their own problems.

#### Potentially Traumatic Events

Children in our sample experienced on average 3.89 (*SD* = 1.97) potentially traumatic events (PTEs). More than half of the children experienced at least four PTEs. The most frequently endorsed experience was (temporary) separation of the parents, which occurred in 86 % of the families.

#### Levels of children’s Post-Traumatic Stress Symptoms

Clinical levels of PTS symptoms were reported in 21 % of all children, based on parent-rated symptoms on the TSCYC. Compared to the norm population (Briere et al. 2001), children in our study scored significantly higher on PTS symptoms (mean T-score of 49 compared to 60, *t*(119) = 8.41, *p* < .001). Table 1 shows the zero-order correlations among PTS symptoms and the other study variables. Children’s PTS symptoms correlated significantly and positively with IPV severity, child abuse, child neglect, and parenting stress. No significant associations were found between children’s PTS symptoms and IPV chronicity, children’s emotional security, and other potential traumatic events.

**Table 1:** Zero-order correlations among study variables

Variable	1.	2.	3.	4.	5.	6.	7.	8.
1. IPV chronicity	−							
2. IPV severity	−.10	−						
3. Traumatic events	−.21^*^	.10	−					
4. Child abuse	.13	.33^**^	−.01	−				
5. Neglect	.01	.25^**^	.07	.31^**^	−			
6. Emotional security	−.09	.02	−.13	.07	.07	−		
7. Parenting stress	−.01	.05	.07	.23^*^	.15	.06	−	
8. PTS symptoms	−.01	.19^*^	.15	.30^**^	.29^**^	.02	.40^***^	−

*IPV* interparental violence, *PTS* post-traumatic stress

^*^ *p* < .05; ^**^ *p* < .01; ^***^ *p* < .001

#### Association of Demographic Variables

We tested whether the demographic variables of child age, child gender, parent ethnicity, and family income were associated with children’s PTS symptoms. Younger age and female gender showed significant associations with children’s PTS and were, therefore, included as control variables in the subsequent hierarchical regression analyses (see Table 2). Ethnicity and family income were not significantly associated with PTS. We also examined the role of time since IPV exposure as a control variable. Time since exposure was not significantly associated with PTS symptoms.

**Table 2:** Hierarchical linear regression models examining trauma and family functioning on post-traumatic stress symptoms (*N* = 120)

	Model 1			Model 2			Model 3		
Variable	*Β*	*SE*(*B*)	*β*	*Β*	*SE*(*B*)	*β*	*Β*	*SE*(*B*)	*β*
Demographics									
Age	−3.74	1.11	−.29^**^	−3.50	1.01	−.27^**^	−3.56	1.01	−.27^**^
Female	7.87	2.47	.27^**^	6.88	2.44	.24^**^	6.88	2.27	.24^**^
Trauma									
IPV chronicity				.12	.21	.05	.14	.20	.06
IPV severity				.01	.18	.01	.05	.17	.02
Traumatic events				1.19	.62	.16	1.00	.59	.14
Child abuse				.77	.35	.20^*^	.47	.33	.12
Neglect				1.86	.97	.17	1.51	.91	.13
Family functioning									
Emotional security							−.05	.27	−.01
Parenting stress							.21	.05	.34^***^
*R* ^*2*^		.16			.27			.38	
*F* for change in *R* ^*2*^		11.05^***^			3.53^***^			9.35^***^	

*IPV* Interparental violence

^*^ *p* < .05; ^**^ *p* < .01; ^***^ *p* < .001

### Association of IPV and Other Experiences With PTS

Hierarchical multiple regression analyses were used to examine the associations of IPV severity and IPV chronicity, child abuse and neglect, and other potential traumatic experiences on PTS symptoms. First, significant associations were found for age and gender, with younger children and girls displaying more PTS symptoms than older children and boys (see Table 2, Model 1). In Model 2, we examined the associations with different types of potentially traumatic experiences (IPV, child abuse, child neglect, and other potentially traumatic events) in step 2 after controlling for age and gender in step 1. The addition of these types of trauma significantly increased the explained variance in PTS symptoms (∆*R*^2^ = .12, ∆*F*(5, 112) = 3.53, *p* = .005). Child abuse was significantly and positively associated with children’s PTS symptoms, whereas the unique associations of IPV severity and chronicity, child neglect, and other potentially traumatic experiences with PTS were not significant (see Table 2, Model 2).

### Association of Family Functioning With PTS

In the third model, we examined the main effects of family functioning, indexed by children’s feelings of emotional security and parenting stress, on PTS symptoms. After accounting for age and gender in step 1, and different types of traumatic experiences in step 2, the addition of emotional security and parenting stress led to a significant improvement of the model (∆*R*^2^ = .11, ∆*F*(2, 110) = 9.35, *p* < .001). Parenting stress was significantly and positively associated with children’s PTS symptoms, whereas no association was found for children’s feelings of emotional security and PTS (see Table 2, Model 3). In the fourth model, the moderating role of parenting stress and emotional security on the association between trauma exposure and PTS symptoms was explored. Therefore, we included 10 interactions between all types of trauma (IPV severity, IPV chronicity, child abuse, child neglect, and other potentially traumatic events) with parenting stress and all types of trauma with children’s feelings of emotional security in step 4, after entering age and gender in step 1, different types of potentially traumatic events in step 2, and main effects of children’s feelings of emotional security and parenting stress in step 3. All variables were centered prior to computing interaction terms. The addition of interaction terms did not significantly improve the model (∆*R*^2^ = .08, ∆*F*(10, 100) = 1.46, *p* = .16). Therefore, the final model included the main effects only. This model is presented in Table 2, Model 3.

## Discussion

The current study examined the impact of interparental violence (IPV) characteristics, child abuse and neglect, and stressful events and explored the moderating role of family functioning on children’s post-traumatic stress (PTS) symptoms in a sample of families who were enrolled for a community-based intervention after experiencing IPV. Consistent with previous literature on trauma and IPV (Alisic et al. 2014; Crusto et al. 2010; Lamers-Winkelman et al. 2012), rates of children’s clinical PTS symptoms (21 %) were considerable in our sample. We first tested the relationship between traumatic childhood experiences, such as IPV, abuse, neglect, and other potential traumatic events and PTS symptoms. According to our results, these experiences together explained 27 % of the variance in trauma symptoms in IPV-exposed children. Second, family functioning indicated by parenting stress contributed independently to PTS symptoms, such that higher perceived stress in the parental role was associated with higher levels of children’s symptoms. Third, we explored whether effects of traumatic experiences on symptoms were moderated by the effects of family functioning. No conditional effect of family functioning on the association between traumatic experiences and PTS symptoms was found for both parenting stress and children’s feelings of emotional security.

In contrast with other studies that documented associations between IPV severity and child internalizing and externalizing problems (Kitzmann et al. 2003), we did not find that severity of IPV uniquely increased the likelihood of children’s PTS symptoms. However, our findings confirmed similar results in a different sample of referred Dutch IPV-exposed children (Lamers-Winkelman et al. 2012). Further, no evidence was found for an association between IPV chronicity and PTS symptoms, even when controlling for age. This lack of effect of chronicity can probably be explained by the observation that the IPV generally lasted for a long time in our sample, given that more than half of our children were exposed to IPV for eight years or longer, which means it could have been present their entire lives. In this way, the exposure to trauma may reflect a chronic condition more than exposure to discrete events (Finkelhor et al. 2007).

In the families that participated in this study, the violence had occurred in the past. Although a meta-analysis has previously provided support for an inverse relationship between time elapsed after a traumatic experience and PTS symptoms (Trickey et al. 2012), we did not find any effects of time since IPV exposure on trauma symptoms, also when controlling for age. However, estimates in the meta-analysis by Trickey et al. (2012) were mainly based on single-event trauma studies and did not include more chronic “condition” types of experiences to which the children in our study were exposed. When children are exposed to chronic interparental violence, spontaneous recovery may be less likely than after exposure to a single traumatic event.

We further examined whether other potential childhood traumatic experiences would be associated with PTS in children exposed to IPV. Consistent with previous work (Lamers-Winkelman et al. 2012), many children in our study were exposed to multiple risk factors, such as child physical and verbal abuse and other potentially traumatic experiences (PTEs). It is well documented that child abuse often co-occurs in IPV families, and multiple stressful experiences tend to cluster within families (Turner et al. 2012). Not only did child abuse correlate with IPV characteristics in our sample, it was also significantly associated with children’s PTS symptoms. Our findings are therefore partly in line with previous work that suggested children who experience multiple adversities are most at risk for poor adjustment (Finkelhor et al. 2007; Finkelhor et al. 2009; Finkelhor et al. 2013; Turner et al. 2012). Similarly, Graham-Bermann et al. (2012) found higher rates of PTS in IPV-exposed children when they were exposed to additional traumatic events.

Finally, we examined whether two indicators of family functioning in the aftermath of IPV could confer additional risk or act as a protective factor on children’s PTS symptoms. First, we examined the role of parenting stress. Parenting stress showed a significant positive relationship with children’s PTS, even after controlling for IPV and other traumatic experiences, highlighting the importance of this factor. This is in line with our hypothesis and previous work on PTS symptoms in violence-exposed children (Crusto et al. 2010), as well as with studies on children’s general psychological adjustment (Crnic and Low 2002). However, we did not find a moderating role of parenting stress in relationships between IPV, abuse, neglect, or potential traumatic experiences and children’s PTS. This is in disagreement with the findings by Levendosky and Graham-Bermann (1998), who reported conditional effects of parenting stress and family violence on internalizing and externalizing problems. Given that caregiver behaviors can have a strong influence in determining a positive or negative outlook in children exposed to trauma (Lieberman and Knorr 2007), more research is needed that further delineates the effects of family functioning on PTS symptoms in the aftermath of IPV.

Our second indicator of family functioning was derived from the Emotional Security Theory (Cummings and Davies 2011). We were interested in whether children’s current perceived emotional security in their family could serve as a buffer against PTS symptoms; however, this was not confirmed. These results are in disagreement with previous studies on the role of support from family members (McCloskey et al. 1995; Miller et al. 2014). Given the potential varying roles of different family members as either a source of support and security or lack thereof, the difference in results may be explained by asking children to assess their security across family members instead of for each member (McCloskey et al. 1995) or by not counting the number of family members in the household (Miller et al. 2014). On the other hand, Forman and Davies (2005) obtained associations with child adjustment in families varying in marital conflict, when controlling for emotional security in specific family subsystems. Furthermore, children’s emotional security at time of the violence might have a greater impact on the association between IPV and PTS symptoms than their emotional security when the threat of violence has ended. The concept of emotional security in the family may need further refinement to understand how emotional security develops when violence in the family has stopped.

### Strengths and Limitations

The current study emphasizes the importance of child abuse and parenting stress in the development of PTS symptoms in children after exposure to IPV. Further, the substantial amount of children suffering from PTS symptoms in our sample underlines that children’s mental health can be severely affected by IPV and highlights the need to assess these symptoms in both research and interventions that target violence-exposed and/or abused children. However, the results of this study need to be interpreted in light of some limitations. First, the correlational design of the study does not allow us to draw conclusions about causality or direction of effects. Thus, we do not know whether parenting stress negatively affects PTS symptoms, or whether PTS symptoms in children evoke more parenting stress, for example. Second, pre-trauma variables could not be taken into account because the families were assessed after they had experienced IPV. Prospective studies addressing these issues are needed but currently lacking. Third, in this study, we do not have information on children’s accounts of the violence, making it impossible to draw conclusions about the child’s own perspective of the events. Given the importance of subjective experience of traumatic events in PTSD (American Psychiatric Association 2013), the child’s own experience of IPV needs to be addressed in subsequent studies. Finally, because we examined our research questions in a referred, high-risk sample that was recruited from community mental health centers, we have likely targeted violence exposure at the more extreme end of the continuum. Therefore, the absence of significant associations between IPV and PTS in our high-risk sample could be reflective of a narrow distribution of exposure and symptoms. Further, the families that were involved in our study were not only more likely to have experienced more serious levels of IPV, but they also experienced multiple risk factors (e.g., poverty and single parenthood). These factors could be a consequence of IPV occurrence; however, given the cross-sectional nature of the sample, it is not possible to establish this timeline. In sum, our findings should be interpreted in light of the limitations of our high-risk sample.

Despite these limitations, our study points out the role of parenting stress as an important factor in PTS symptomatology of children exposed to violence. Given the limited body of research on children’s PTS symptoms in violent families, this study highlights the importance of considering aspects of co-occurring traumatic and stressful events as well as family functioning in trauma-informed care for children exposed to IPV.

### Conclusions and Implications

Our findings emphasize the polyvictimization that occurs in IPV exposed children, consistent with previous work that suggests IPV rarely occurs in isolation (Turner et al. 2012). More importantly, our study also underscores the importance of addressing current parenting stress as an important risk factor in the development of PTS. Our findings have implications for the treatment of violence-exposed children. First, they underscore the importance of trauma-informed care, in that a substantial amount of children in our study experienced clinical levels of trauma symptoms. Second, this trauma-informed approach should encompass an assessment of traumatic experiences not limited to IPV. Such an assessment should take into account the possibility of polyvictimization, such as child abuse as well as other potentially traumatic experiences. Finally, it is important to attend to the needs of the IPV-exposed parent and attempts should be made to alleviate parenting stress. Recently, more attention has been given for developing effective treatments for IPV-exposed children (Rizo et al. 2011), and studies on the effectiveness of treatments that actively involve parents, or include parenting components in this group, or both (e.g., Parent–Child Interaction Therapy; Kid’s Club; Child-Parent Psychotherapy) are limited but show promising results (Graham-Bermann et al. 2007; Jouriles et al. 2001; Lieberman et al. 2005).
